# Idiopathic Neonatal Colonic Perforation

**Published:** 2014-01-01

**Authors:** Nihat Demir, Oğuz Tuncer, Mehmet Melek, Sultan Kaba, Keziban Bulan, Erdal Peker

**Affiliations:** 1 Department of Pediatrics, Yuzuncu Yil University School of Medicine, Van, Turkey; 2 Department of Pediatric Surgery, Yuzuncu Yil University School of Medicine, Van, Turkey

**Keywords:** Sigmoid colon, Perforation, Neonate

## Abstract

Though the perforation of the colon in neonates is rare, it is associated with more than 50% mortality in high-risk patients. We report a case of idiopathic neonatal perforation of the sigmoid colon in an 8-day-old, healthy, male neonate without any demonstrable cause.

## INTRODUCTION

Introduction Though the perforation of the colon in neonates is rare, it is associated with more than 50% mortality in high-risk patients [1]. Idiopathic perforation of the bowel, also known as spontaneous intestinal perforation (SIP), occurs without any demonstrable cause. Colonic perforation in very-low-birth-weight (VLBW) infants may be caused by various conditions such as necrotizing enterocolitis (NEC), Hirschsprung's disease (HD), mechanical obstructions, meconium plug syndrome, small left colon syndrome, idiopathic perforations, isolated intestinal perforation, stercoral perforations and rarely cystic fibrosis [2, 3]. SIP in VLBW infants have been reported as a distinct pathologic entity from NEC. Nonetheless, some clinical features may be significantly different between NEC and SIP [4]. We report a case of idiopathic perforation of the sigmoid colon in a neonate.

## CASE REPORT

A 1350-g male child of 32 weeks gestation age was born to a 25-year-old mother by spontaneously vaginal delivery. Apgar scores were 6 and 9 at 1 and 5 min, respectively. He did not require artificial ventilation support. The post-natal period was uneventful till day 8, and enteral feeding had been started successfully. On the 8th day of life, he presented with a distended abdomen but soft and lax, without any redness of abdominal wall. Although he had bowel sounds in this process, but stool was not passed. Abdominal X-rays showed distended intestinal loops and a marked pneumoperitoneum bilaterally. Clinical diagnosis of perforation peritonitis was done at this instance and his enteral feeding was stopped along with insertion of NG tube. Biochemical examinations were normal. Total parenteral nutrition and antibiotics were started. Exploratory laparotomy revealed meconium peritonitis and a focal perforation on the 1/3 distal part of sigmoid colon (0.5x0.5 cm, the size of perforation range) (Fig. 1). Full-thickness rectal biopsy on frozen section examination was reported to have normal ganglion cells. The colon size was normal. Margins of the perforation were freshed and closed primarily. Histopathological examination of the margins of perforation was normal. The patient recovered promptly. He is doing fine on follow-up.

**Figure:**
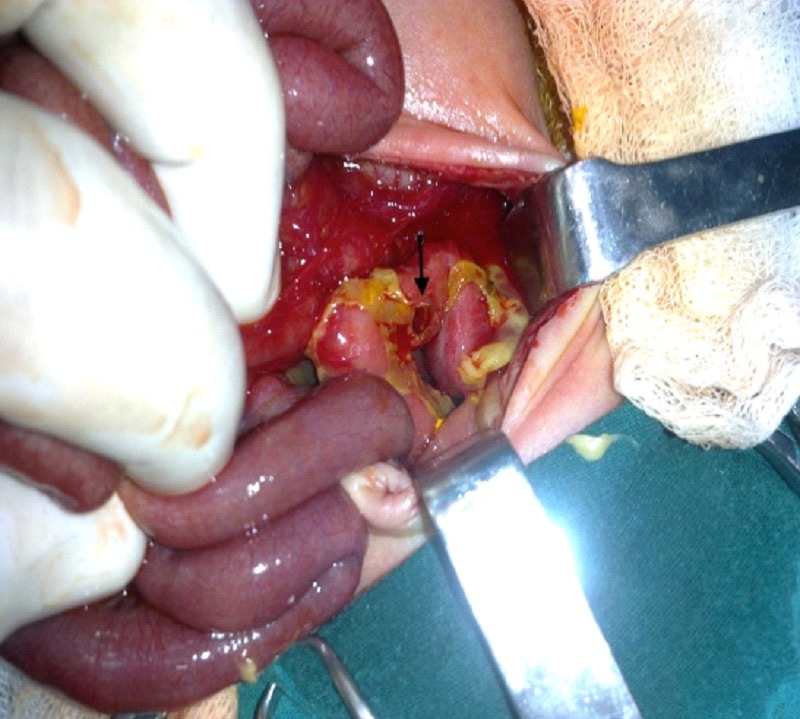
Figure 1: Focal perforation site on the 1/3 distal part of sigmoid colon

## DISCUSSION

SIP was first described by Breslau in 1863. It accounts for one-third of all colonic perforations in neonates [1]. The most important etiologic factors are umbilical arterial catheterization, use of indomethacin, cytomegalovirus and coagulase negative staphylococcal infections, congenital structural abnormalities of the intestine, trauma, and mechanical obstruction [5, 6, 7]. There was none of these etiologic factors in our case. Although viral serology cannot be performed, blood culture was negative, and histopathological examinations were normal. 

There could only be speculation about the cause of perforation in these infants. Some authors suggest that ischemia is the common etiologic factor [8], but this etiology may clearly be related to hypoxic neonates. Another problem common seen in VLBW infants is immaturity of the intestinal motility. Resch et al, speculated the combination of episodes of hypoxia, resulting in ischemic injury to the bowel, together with immaturity of intestinal motility could result in SIP in VLBW infants [9]. But in our patient the features suggestive of these condition were absent.

Colonic perforation that is unknown causation also occurs in the VLBW infants. Many factors may lead to the development of idiopathic colon perforation. Invasion of the intestinal wall by coliform bacteria and local Schwartzman reaction excited by endotoxins of bacteria were proposed as possible mechanisms for idiopathic colonic perforation in newborn infants [10, 11]. Many cases of idiopathic perforation of colon in VLBW infants, without evident demonstrable reason, may be due to pseudo obstruction of the left colon. Small left colon syndromes in infants are usually born to diabetic mothers. The mother of our patient didn't have diabetes and both proximal and distal colon was normal. 

Patients with SIP present earlier and seem to have a better prognosis than NEC. Most patients with NEC have been previously fed via the enteral route. Although NEC does occur in unfed premature infants, SIP, a distinct pathologic entity, commonly presents in ELBW infants during the first couple of weeks after birth and before the initiation of feeding (Table 1) [12].

**Figure:**
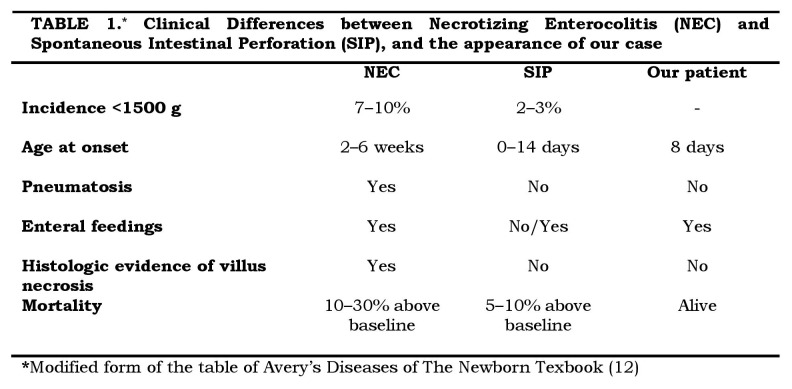
Table 1: Clinical Differences between Necrotizing Enterocolitis (NEC) and Spontaneous Intestinal Perforation (SIP), and the appearance of our case

In preterm babies, pneumoperitoneum is mostly due to NEC; however, in full-term babies and early infancy, the etiology is quite different. Singh and colleagues evaluated 60 full-term newborns with colonic perforation; they reported that in 78.33% of the patients, Hirschsprung's disease (HD) is responsible for the etiology of colonic perforations. They recommend biopsy in all perforations to differentiate between NEC, SIP, and HD [1]. In our case biopsies were reported normal so we dealt this perforation by primary repair rather than a stoma.

## Footnotes

**Source of Support:** Nil

**Conflict of Interest:** None

